# Editorial: Perforins and Cholesterol-Dependent Cytolysins in Immunity and Pathogenesis

**DOI:** 10.3389/fimmu.2022.932502

**Published:** 2022-06-09

**Authors:** George P. Munson, Robert J. C. Gilbert, Gabriele Pradel

**Affiliations:** ^1^ Department of Microbiology and Immunology, Leonard M. Miller School of Medicine, University of Miami, Miami, FL, United States; ^2^ Division of Structural Biology, Wellcome Centre for Human Genetics, University of Oxford, Oxford, United Kingdom; ^3^ Cellular and Applied Infection Biology, RWTH Aachen University, Aachen, Germany

**Keywords:** perforin-2/MPEG1, membrane attack complex (MAC), pore-forming protein, complement, cholesterol-dependent cytolysins (CDC), plasmodium perforin like proteins (PPLP), pneumolysin (PLY), suilysin (SLY)

In humans and other mammals, the pore-forming proteins C9 and the perforins are important innate immune effectors. C9 forms the membrane attack complex (MAC) in an assemblage with the complement proteins C5b, C6, C7, and C8 that attacks the envelopes of gram-negative bacteria. Perforin is deployed by degranulating killer lymphocytes to destroy virally infected or cancerous cells. The product of macrophage expressed gene 1 (Mpeg1), named perforin-2 by the late Eckhard R. Podack (1943–2015), appears to play a central role in the destruction of phagocytosed bacteria. All three pore-forming proteins contain a membrane attack complex-perforin (MACPF) domain. However, the MACPF domain is not exclusive to mammals. Rather, proteins with MACPF domains are found in a diverse array of taxa including plants, fungi, invertebrates, and a variety of protozoans such as the malaria parasite *Plasmodium falciparum*, or *Toxoplasma gondii*, the causative agent of toxoplasmosis. MACPF domains have also been identified in some prokaryotes including pathogenic species of *Chlamydia*. Pore-forming proteins within the MACPF family share a canonical fold with the cholesterol-dependent cytolysins (CDCs) (1). CDCs are pore-forming proteins that contribute to the virulence of several gram-positive pathogens such as *Clostridium perfringens*, *Listeria monocytogenes*, and group A streptococci. Thus, evolution has produced pore-forming proteins with opposing roles in host defense and pathogenesis that have shared structural features. As previewed below, this article collection highlights the immunological and pathological roles of MACPF/CDC pore-forming proteins.

## Perforin-2: An Ancient Pore Forming Protein

Perforin-2 (MPEG1) is the most recently described member of the MACPF family of pore forming proteins. Although newly introduced by researchers in the fields of immunology and structural biology, perforin-2 is an evolutionarily ancient protein that spans taxa from sponges to humans (2). Two reviews focus on perforin-2 and each provides a unique perspective on the topic. Both reviews critically evaluate the current literature and discuss areas that warrant further investigation to resolve discrepancies and uncertainties. The review by Bayly-Jones et al. emphasizes the structural features and molecular mechanisms of pore formation by mammalian perforin-2). Although mammalian perforin-2 is also discussed in the review by Merselis et al., the authors also evaluate studies of perforin-2 in invertebrates and bony fish. The discussion of invertebrate perforin-2 is complemented by a perspective by Walters et al. that covers the evolution and possible roles of perforin-2 in corals, sea anemones and other Cnidarians.

The research article by Pastar et al. investigates a connection between a commensal skin microbe and perforin-2. The investigators find that *Staphylococcus epidermidis* activates gamma delta T cells and increases the expression of perforin-2 in gamma delta T cells, keratinocytes, and fibroblasts. Interestingly, this may be beneficial to the host because the induction of perforin-2 enhances the killing of intracellular *Staphylococcus aureus* by skin cells. In a related minireview by O’Neill et al., the authors expand upon the findings of Pastar et al. by reviewing the roles of both perforin and perforin-2 in gamma delta T cells.

Merselis et al. present the first case report of perforin-2 deficiency. They report the case of a female patient with recurrent abscesses and cellulitis of the breast that was refractory to antibiotic treatment and surgical interventions. Whole genome sequencing revealed a nonsense mutation, Tyr430*, in one allele of *mpeg1* resulting in a severely truncated protein. Functional analyses find that the patient’s macrophages and neutrophils were less able to kill intracellular bacteria than cells from healthy donors. This case report of perforin-2 haploinsufficiency and the study by Pastar et al. establish the importance of perforin-2 in skin and soft tissues.

Although perforin-2 is primarily thought of as an innate immune effector, the research article by Frasca et al. demonstrates that perforin-2 deficiency also affects adaptive immunity. Specifically, the researchers find that perforin-2 knockout mice have reduced antibody responses to an influenza vaccine compared to wild-type mice. This is due to chronic inflammation of knockout mice caused by their failure to efficiently block the translocation of gut microbes and/or microbial products to extra-intestinal sites. Systemic, but low-grade, inflammation causes intrinsic B cell inflammation and reduces their ability to produce antibodies in response to antigens. Thus, the researchers demonstrate a previously unknown connection between perforin-2 and a properly functioning adaptive immune response.

## Soluble Complexes of MACPFs and the Prevention of Off-Target Effects

The MAC of the complement system has been extensively characterized at immunological, mechanistic, and molecular levels. In contrast, the soluble MAC (sMAC) is much less understood. Both are assemblages of the complement proteins C5b, C6, C7, C8, and C9, but the stoichiometry of complement proteins in MAC and sMAC are radically different. For example, sMAC may also contain clusterin and/or vitronectin, and unlike MAC are not pore forming complexes. These and other differences are reviewed by Barnum et al. The authors also discuss sMAC as a long standing enigma in the field of immunology. Nevertheless, the authors provide a compelling case for the utility of sMACs as biomarkers of disease.

Although the pore forming proteins MAC, perforin, perforin-2, and gasdermins have important defensive and immunological functions, their capacity to form pores in lipid bilayers poses a risk of self-inflicted harm to the host. This biological conundrum is addressed in the review by Krawczyk et al. Their review covers the “where and when” of pore-forming protein expression, processing, subcellular storage, and molecular mechanisms that prevent the attack of off-target membranes.

## Perforin-Like Proteins in Protozoan Parasites

Perforin-like proteins were also identified in apicomplexan parasites, intracellularly-living protists that actively invade their host cells. These parasites require a fine-tuned molecular machinery for host cell penetration and egress, but also for crossing epithelial barriers, and some of these functions are mediated by perforins. In their review article, Sassmannshausen et al. highlight the diverse roles of the perforin-like proteins during life-cycle progression of apicomplexan parasites. The authors lay the focus on the two parasites *Plasmodium* and *Toxoplasma*, the causative agents of malaria and toxoplasmosis, for which the perforins are best studied. In these parasites, perforins are used to perforate the membranes of the parasitophorous vacuole and the host cell membrane during exit, but also to overcome epithelial barriers during tissue passage. *Plasmodium* and *Toxoplasma* express five and two variants of perforins, respectively, and these perforins appear to have distinct functions that are specific for both tissue type and life-cycle stage.

In this context, the original research article by Garg et al. reports on the pore-forming activity of the Pan-active MACPF Domain (PMD), a centrally located and highly conserved region of perforin-like proteins of the malaria parasite, and further evaluate the inhibitory potential of specifically designed PMD inhibitors. The authors show that the incubation of erythrocytes with PMD induces senescence in these cells, which may attribute to severe malarial anemia. Anti-PMD inhibitors effectively prevent the parasites from invasion of, and egress from, host erythrocytes, but also block the hepatic stages and transmission stages of the malaria parasite, suggesting that PMDs represent multi-stage, transmission-blocking inhibitors.

## CDCs and MACPFs of Bacterial Pathogens

The research article by Song et al. elucidates the contribution of suilysin to the development of streptococcal toxic shock-like syndrome. Suilysin is a CDC produced by *Streptococcus suis*. Although primarily a swine pathogen of significant concern to the agricultural industry, *S. suis* can also cause meningitis and streptococcal toxic shock-like syndrome in humans. Through a combination of *in vitro* and *in vivo* approaches the authors show that suilysin causes the release of IL-1β *via* activation of the NLRP3 inflammasome. Although the mechanism of inflammasome activation is currently unknown, chemical or genetic blockade of the pathway protects mice from suilysin’s cytotoxic effects.

*Streptococcus pneumoniae* is a commensal microbe that commonly colonizes the nasal cavity. Although most colonized individuals are asymptomatic, *S*. *pneumoniae* is also an opportunistic pathogen capable of causing a wide range of symptoms and disease outcomes. The minireview by Nishimoto et al. discusses the role of pneumolysin in various phases of pneumococcal disease. Pneumolysin is a CDC that is cytolytic to a wide range of host cell types that contributes to both transmission and colonization. The authors conclude with a review of vaccines against the CDC and drugs that limit pneumolysin’s cytotoxic effects.

Unlike pneumolysin, the contribution of hemolysin to *Vibrio vulnificus* pathogenicity has been more controversial. *V. vulnificus* infections are typically acquired through the consumption of raw shellfish and in some cases can lead to fatal septicemia. The minireview by Yuan et al. evaluates recent progress towards understanding the biological effects of the *V. vulnificus* CDC and its roles in gastroenteritis and septicemia.

The review by Keb and Fields returns the discussion to MACPFs. *Chlamydia* spp. are obligate intracellular bacteria that parasitize eukaryotic cells. The authors review evidence that suggest *Chlamydia* pathogenicity is little impacted by MAC. They propose that this can be explained by the unique properties of the envelopes of extracellular elementary bodies that likely make them refractory to the deposition of the complement proteins of MAC. Also reviewed are studies indicating that Perforin of natural killer cells and cytotoxic T lymphocytes is not a major mediator of *Chlamydia* infectivity and pathogenicity. In contrast to MAC and Perforin, the authors review evidence that indicates perforin-2 significantly limits chlamydial infection. They conclude with a discussion of chlamydial MACPFs.

## Perivitellin-2 Enterotoxin: An Unusual Evolutionary Adaptation of a MACPF

Undoubtedly, the most unexpected submission to the collection was the research article by Giglio et al. *Pomacea maculata* is a snail species whose eggs contain a toxin known as perivitellin-2 (PV2). This toxin contains both a lectin binding domain and a MACPF, and the authors show that PV2 is cytotoxic to immune and epithelial cells. The latter are significant because the authors propose that PV2 may have evolved to prevent predation of the eggs. As predicted, PV2 is found on the surface of enterocytes and induces morphological changes in the small intestines of mice orally challenged with PV2. Based on several criteria the authors argue that PV2 meets the definition of an enterotoxin and, as such, it is the first known animal enterotoxin.

## Conclusions

Although unified by the topic of perforins and CDCs, this collection brings together diverse viewpoints from researchers from around the globe. This diversity is reflected by the variety of subject matter in articles that span from mini-reviews to original research articles. Authors represent numerous disciplines and specialties including, but not limited to, structural and evolutionary biology, parasitology, bacterial pathogenesis, and immunology. We thank them for their contributions and participation in this endeavor. Their unique perspectives, experimental approaches, and critical evaluations of the literature have come together in this collection to provide a broad understanding of the field of MACPF/CDCs.

## Author Contributions

GM and GP composed the first draft of the manuscript. All authors reviewed the document and contributed to editing. All authors contributed to the article and approved the submitted version.

## Funding

Perforin-2 research in the laboratory of GM is supported by the National Institute of Allergy and Infectious Diseases of the National Institutes of Health under award numbers R01AI110810, R21AI156567, and R21AI159794. The content of this publication is solely the responsibility of the authors and does not necessarily represent the official views of the National Institutes of Health. GP acknowledges funding by the SPP 2225 of the Deutsche Forschungsgemeinschaft (PR905/19-1).

## Conflict of Interest

The authors declare that the research was conducted in the absence of any commercial or financial relationships that could be construed as a potential conflict of interest.

## Publisher’s Note

All claims expressed in this article are solely those of the authors and do not necessarily represent those of their affiliated organizations, or those of the publisher, the editors and the reviewers. Any product that may be evaluated in this article, or claim that may be made by its manufacturer, is not guaranteed or endorsed by the publisher.
